# Efficacy and safety of oliceridine versus sufentanil for patient-controlled intravenous analgesia following gynecological laparoscopic surgery: a randomized, double-blind, parallel-group trial

**DOI:** 10.3389/fmed.2026.1866393

**Published:** 2026-08-11

**Authors:** Jingmei Zhou, Zhuangbo Wu, Yuqing Lai, Jingxin Shi, Wenbo Chen, Zhengqin Wu, Xukeng Guo, Meizhen Li, Xingcong Jiang, Shaohui Zhuang, Ronghua Huang, Weiqi Ke

**Affiliations:** 1 Department of Anesthesiology, The First Affiliated Hospital of Shantou University Medical College, Shantou, Guangdong, China; 2 Department of Anesthesiology, Shantou University Medical College, Shantou, Guangdong, China

**Keywords:** biased *μ*-opioid receptor agonist, enhanced recovery after surgery, laparoscopic surgery, oliceridine, patient-controlled intravenous analgesia, postoperative nausea and vomiting

## Abstract

**Background:**

To compare the efficacy and incidence of adverse reactions of oliceridine versus sufentanil in patient-controlled intravenous analgesia (PCIA) for postoperative pain management following gynecological laparoscopic surgery.

**Methods:**

In this randomized, double-blind trial, 152 patients undergoing elective gynecological laparoscopic surgery were randomly assigned to receive PCIA with either oliceridine (Group O, 0.4 mg/kg, n = 76) or sufentanil (Group S, 2 μg/kg, n = 76). The primary outcome was the Visual Analog Scale (VAS) score at rest at 2, 4, 8, 12, 18, 24, 36, and 48 h postoperatively. Secondary outcomes included postoperative Mini-Mental State Examination (MMSE) scores, number of PCIA demands, rescue analgesic use, adverse events, time to first flatus and defecation, patient satisfaction, and length of hospital stay.

**Results:**

Resting VAS scores were comparable between groups except at 4 h (*p* < 0.05). No significant differences were observed in MMSE scores, PCIA demands, rescue analgesia, time to first defecation, or other adverse events (pruritus, dizziness, fever, headache, cough, and abdominal distension) (all *p* > 0.05). Group O had higher body weight and body mass index (BMI) than Group S (*p* = 0.028 and *p* = 0.041, respectively). Group O demonstrated lower postoperative nausea and vomiting (PONV) incidence (19.48% vs. 36.14%; relative risk (RR), 0.54; 95% confidence interval (CI), 0.32–0.92; *p* = 0.019), prolonged time to first flatus (27.16 ± 9.33 h vs. 23.23 ± 9.36 h; *p* = 0.014), and higher patient satisfaction (4.09 ± 0.81 vs. 3.74 ± 0.72; *p* = 0.006).

**Conclusion:**

For postoperative analgesia following gynecological laparoscopic surgery, oliceridine provides effective analgesia comparable to sufentanil while significantly reducing the risk of opioid-related PONV, demonstrating a favorable safety profile.

**Clinical trial registration:**

http://www.chictr.org.cn/, ChiCTR2400089648.

## Introduction

Inadequate postoperative pain management remains a universal clinical challenge. Despite the availability of various analgesic modalities, approximately 75% of surgical patients experience postoperative pain, with the majority reporting moderate-to-severe intensity (1, 2). Uncontrolled acute pain not only compromises patient satisfaction but also precipitates a cascade of adverse physiological consequences, including cardiovascular complications, respiratory depression, gastrointestinal dysfunction, immunosuppression, and postoperative cognitive dysfunction (POCD), ultimately prolonging hospitalization, increasing healthcare costs, and potentially progressing to chronic pain, thereby severely impairing long-term quality of life (3–6). Effective postoperative analgesia constitutes a cornerstone of Enhanced Recovery After Surgery (ERAS) protocols and is pivotal to improving patient satisfaction (7, 8).

For decades, *μ*-opioid receptor agonists (e.g., sufentanil) have served as the mainstay of postoperative analgesia owing to their potent analgesic efficacy (3, 9). However, their analgesic effects and adverse reactions are both mediated by *μ*-opioid receptors—the analgesic effect arises from G-protein pathway activation, whereas dose-dependent adverse reactions such as respiratory depression (opioid-induced respiratory depression, OIRD) and PONV are closely associated with *β*-arrestin pathway activation (10) and represent major clinical challenges (11, 12). This mechanistic overlap limits the safe application of traditional opioids, particularly in high-risk populations such as females and non-smokers (13–15). Therefore, the search for alternative agents capable of dissociating analgesia from adverse effects represents a critical unmet need in improving postoperative pain management.

Oliceridine (Oliceridine, TRV130) is a novel G-protein-biased *μ*-opioid receptor agonist that achieves dissociation of analgesia from adverse effects through selective activation of G-protein signaling pathways while minimizing *β*-arrestin recruitment (16, 17). Preclinical and clinical studies have demonstrated that, compared with equianalgesic doses of morphine, oliceridine significantly reduces the incidence of OIRD (18, 19), while also exhibiting reduced gastrointestinal adverse effects compared with conventional opioids (20), and superior neurocognitive safety (21), ultimately exhibiting superior overall safety and tolerability. Oliceridine was approved by the U.S. Food and Drug Administration (FDA) in 2020 for the management of moderate-to-severe acute pain in adults (22, 23). However, no randomized controlled trial has directly compared the efficacy and safety of oliceridine versus sufentanil—a potent traditional opioid widely used in postoperative PCIA in China—in a head-to-head manner, particularly in the specific population undergoing gynecological laparoscopic surgery, which presents unique requirements for both analgesia and PONV management.

Gynecological laparoscopic surgery patients exhibit distinct clinical characteristics: relatively minor surgical trauma but significant visceral traction, carbon dioxide pneumoperitoneum-induced referred shoulder pain, and predominantly female gender—the latter being an independent risk factor for PONV in the Apfel scoring system (female gender, non-smoking status, history of motion sickness, and postoperative opioid use) (13, 15). Consequently, this population demands analgesic regimens that not only provide adequate pain control but also minimize PONV risk to facilitate early feeding, early mobilization, and rapid recovery (8). Although sufentanil provides reliable analgesia, its *β*-arrestin pathway-mediated risks of PONV and respiratory depression are particularly pronounced in these high-risk patients (24, 25), often necessitating combination with multiple antiemetic agents or restriction of analgesic dosing, which paradoxically compromises analgesic quality.

Based on this background, the present study aimed to systematically evaluate the analgesic efficacy and safety of oliceridine versus sufentanil for postoperative PCIA in patients undergoing gynecological laparoscopic surgery through a randomized, double-blind, parallel-controlled clinical trial. We hypothesized that oliceridine would provide non-inferior analgesic efficacy to sufentanil while significantly reducing the incidence of opioid-related adverse reactions, thereby offering an optimal postoperative analgesic option for this specific high-risk population and providing evidence-based support for individualized analgesic strategies within ERAS pathways.

## Methods and participants

### Study design and ethical approval

This single-center, prospective, randomized, double-blind, parallel-controlled clinical trial was conducted at the First Affiliated Hospital of Shantou University Medical College from November 2024 to November 2025. The study protocol was approved by the Ethics Committee of the First Affiliated Hospital of Shantou University Medical College (Approval No. B-2023-236) and registered with the Chinese Clinical Trial Registry (Registration No. ChiCTR2400089648). Written informed consent was obtained from all participants prior to enrollment. The conduct and reporting of this trial adhered to the Consolidated Standards of Reporting Trials (CONSORT) statement.

### Study population

Study participants were adult female patients scheduled for elective gynecological laparoscopic surgery (including laparoscopic hysterectomy, myomectomy, and ovarian cystectomy). Inclusion criteria were: age 18–80 years; body mass index (BMI) 18–30 kg/m^2^; scheduled for gynecological laparoscopic surgery under general anesthesia; American Society of Anesthesiologists (ASA) physical status I-II; and provision of written informed consent after adequate understanding of the study. Exclusion criteria included: known allergy or contraindication to opioid analgesics; history of drug or alcohol addiction; severe hepatic or renal dysfunction [alanine aminotransferase (ALT) or aspartate aminotransferase (AST) > 3 times the upper limit of normal, creatinine clearance <30 mL/min]; severe organic brain disease; New York Heart Association (NYHA) functional class ≥III; coagulopathy [international normalized ratio (INR) > 1.5]; sick sinus syndrome or second-degree or higher atrioventricular block; use of opioid analgesics within 24 h prior to surgery; participation in other drug clinical trials within the past 6 months; pregnancy or lactation; and any other conditions deemed unsuitable for inclusion by the investigators. Additional exclusion criteria after enrollment included: failure to undergo surgery or anesthesia according to the study protocol; occurrence of serious adverse events during study drug administration; persistent pain (VAS score >5) despite rescue analgesia according to the postoperative analgesic protocol; withdrawal of consent by the patient or family members; unplanned postoperative transfer to the intensive care unit (ICU); and failure to cooperate with postoperative follow-up or loss to follow-up.

### Randomization and blinding

This study employed a double-blind design, with research personnel stratified into quality control staff, operating personnel, and follow-up personnel. Patients, surgeons, anesthesiologists responsible for intraoperative and postoperative management, nursing staff, and follow-up personnel were all blinded to group allocation. Eligible participants were randomly assigned in a 1:1 ratio to either the oliceridine group (Group O) or the sufentanil group (Group S) using computer -generated random numbers. Group allocation was concealed using sequentially numbered, sealed, opaque envelopes. During implementation, quality control personnel registered only the group sequence numbers and allocations, instructing operating personnel to execute the therapeutic strategy for analgesic pump drug preparation. The PCIA pumps and solutions used in both groups were identical in appearance, color, and volume to ensure blinding maintenance. Unblinding was permitted only in the event of serious adverse events (e.g., respiratory depression) requiring definitive therapeutic intervention, after which the patient was withdrawn from the study. Follow-up personnel were responsible for data entry. Unblinding was performed at the conclusion of the clinical trial by the project coordinator, quality control personnel, and principal investigator after verification of group allocation information and aggregation of project data for statistical analysis.

### Anesthesia and intervention protocol

On the day prior to surgery, patients received detailed instruction regarding PCIA device usage and the Visual Analog Scale (VAS) until proficient mastery was achieved. Patients were also advised to ambulate as early as possible postoperatively when pain was tolerable.

All patients received standardized general anesthesia. Upon entering the operating room, routine monitoring included non-invasive blood pressure (NIBP), electrocardiography, pulse oxygen saturation (SpO2), end-tidal carbon dioxide partial pressure (etCO2), and bispectral index (BIS). Anesthesia induction was performed with ciprofol 0.4 mg/kg, sufentanil 0.3–0.5 μg/kg, and cisatracurium 0.2 mg/kg. Tracheal intubation was performed after BIS values stabilized within the range of 40–60 for 60 s, followed by connection to an anesthesia machine for volume-controlled ventilation. Ventilation parameters were set as follows: tidal volume 6–8 mL/kg, respiratory rate 10–15 breaths/min, positive end-expiratory pressure (PEEP) 4–8 cmH2O, inspired oxygen concentration 30–50%, maintaining etCO2 between 35 and 45 mmHg, peak airway pressure <30 cmH2O, and SpO2 > 90%.

Total intravenous anesthesia was maintained intraoperatively with continuous infusion of ciprofol 0.4–2.4 mg/(kg·h) and remifentanil 0.1–0.2 μg/(kg·min), maintaining BIS values between 40 and 60. NIBP fluctuations were actively maintained within ±20% of baseline values. Thirty minutes before surgical completion, intravenous sufentanil 0.1 μg/kg was administered as an analgesic transition. At the conclusion of surgery, ondansetron 0.1 mg/kg was administered for PONV prophylaxis, combined with neostigmine 0.04 mg/kg and atropine 0.02 mg/kg for neuromuscular blockade reversal. Patients were encouraged to regain consciousness until eye-opening when BIS >70, followed by tracheal extubation when extubation criteria were met, and subsequent transfer to the recovery room. The PCIA pump was connected and initiated at the time of skin closure.

Drug dosages were determined based on previous clinical studies and pharmacological equivalence analysis. According to data from the APOLLO-1 and APOLLO-2 trials, the analgesic potency of oliceridine is approximately 5 times that of morphine (26, 27), whereas sufentanil is approximately 1,000 times that of morphine (28). Based on equianalgesic dose conversion, 0.4 mg/kg oliceridine is equivalent to 2.0 mg/kg morphine, from which the equianalgesic dose of sufentanil was calculated as 2 μg/kg, indicating comparable analgesic potency between the two agents.

The PCIA protocols were configured as follows:

Group O: oliceridine 0.4 mg/kg + tropisetron 10 mg + 0.9% sodium chloride injection to 150 mL;
Group S: sufentanil 2 μg/kg + tropisetron 10 mg + 0.9% sodium chloride injection to 150 mL.

The PCIA parameters were as follows: background infusion rate 2 mL/h, bolus dose 1 mL, lockout interval 15 min, with no loading dose. When VAS score >5 and PCIA efficacy was inadequate, intravenous tramadol 0.1 g was administered as rescue analgesia, with a minimum interval of 8 h between rescue doses. PCIA was continued until 48 h postoperatively, solution depletion, patient request for discontinuation, or discontinuation due to adverse effects.

### Outcome measures

The primary efficacy endpoint was postoperative pain intensity at rest assessed by the VAS (0 indicating no pain and 10 indicating worst possible pain), evaluated at 2, 4, 8, 12, 18, 24, 36, and 48 h after surgery.

Secondary endpoints included: (1) Postoperative cognitive function, assessed by the MMSE scores at 24 and 48 h postoperatively; (2) PCIA utilization, recording the total number of PCIA demands, effective demands (defined as the first demand within the 15-min lockout interval), and actual administrations within 48 h postoperatively, with increased effective demands indicating inadequately met analgesic requirements; (3) Rescue analgesia requirements, recording the proportion of patients requiring intravenous tramadol (0.1 g) and frequency of use; (4) Incidence of adverse effects, including PONV, pruritus, dizziness, fever (body temperature ≥38 °C), headache, cough, and abdominal distension; (5) Gastrointestinal function recovery, recording time to first flatus and time to first defecation; (6) Patient satisfaction, assessed using a 5-point Likert scale at 48 h postoperatively (1 = very dissatisfied, 2 = dissatisfied, 3 = neutral, 4 = satisfied, 5 = very satisfied).

### Statistical analysis

Statistical analysis was performed using EmpowerStats software (version 5.0) and GraphPad Prism (version 8.0.1).

Based on pilot study results, assuming a between-group difference in resting VAS score at 24 h postoperatively of 0.5 points with a standard deviation of 1.5 points, a two-sided *α* = 0.05, statistical power (1 − *β*) = 0.80, and a non-inferiority margin *δ* = 1.0 point, and considering a 20% dropout rate, PASS software (version 15.0) calculated that approximately 80 patients per group (total 160 patients) were required. Ultimately, 152 patients were enrolled.

Continuous data are presented as mean ± standard deviation (Mean ± SD) or median (interquartile range) [*M* (IQR)], while categorical data are presented as frequency (percentage) [*n* (%)]. Normality was assessed using the Shapiro–Wilk test, and homogeneity of variance using the Levene test. Continuous variables with normal distribution and homogeneity of variance were analyzed using independent samples t-test, reporting Cohen’s d effect size and 95% confidence interval (CI); non-normally distributed variables or those with heterogeneity of variance were analyzed using the Mann–Whitney U test. Repeated measures data (VAS scores) were analyzed using repeated-measures analysis of variance (Greenhouse–Geisser correction), with simple effects analysis performed when interaction effects were significant and Bonferroni correction applied for multiple comparisons. Categorical data were analyzed using *χ*^2^ test or Fisher’s exact test, reporting relative risk (RR) or odds ratio (OR) and 95% CI.

Both intention-to-treat (ITT) and per-protocol (PP) analyses were conducted. Missing data were handled using multiple imputation by chained equations (MICE, m = 5), with sensitivity analysis performed to assess result robustness. All statistical tests were two-sided, with *p* < 0.05 considered statistically significant.

## Results

### Patient screening and group allocation

This study screened 160 patients scheduled for gynecological laparoscopic surgery under general anesthesia. Among these, 5 patients were excluded for not meeting inclusion criteria (3 with BMI > 30 kg/m^2^, 2 with ASA III classification), leaving 155 patients who provided informed consent and were enrolled. Using block randomization, enrolled patients were randomly assigned in a 1:1 ratio to two groups: the oliceridine group (Group O, *n* = 77) and the sufentanil group (Group S, *n* = 78). All randomized patients received the allocated postoperative intravenous patient -controlled analgesia intervention according to the study protocol.

**Figure 1:**
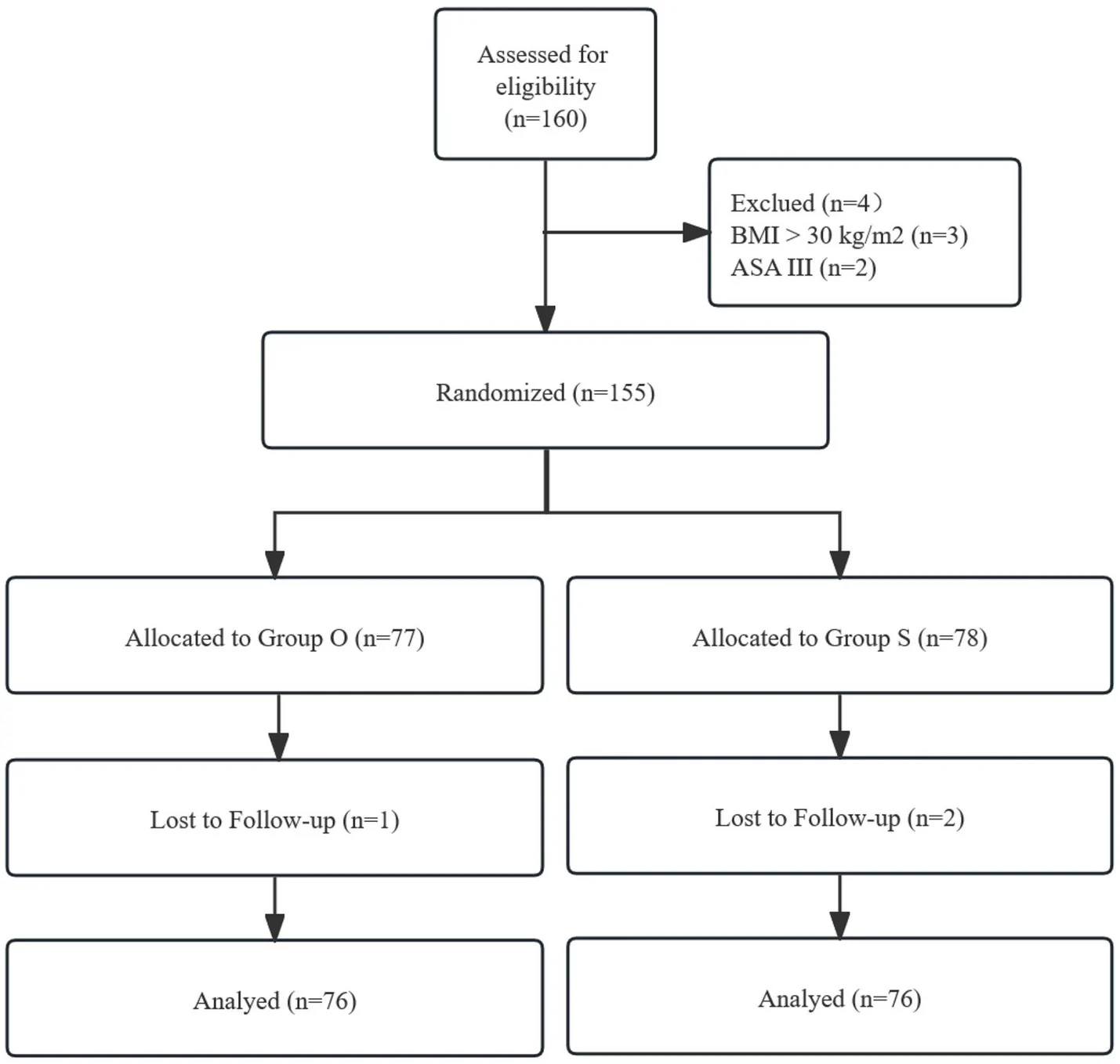
The flowchart of the participants in the study.

During the postoperative follow-up phase, 1 patient in Group O was excluded (refused follow-up), and 2 patients in Group S were excluded (both refused follow-up). Ultimately, data from 152 patients were included in the Full Analysis Set (FAS) for statistical analysis, with 76 patients in each group. The Per-Protocol (PP) analysis included 152 patients. The participant screening, randomization, and follow-up flowchart is detailed in Figure 1 (CONSORT flow diagram).

### Baseline characteristics

The two groups demonstrated excellent comparability in demographic characteristics, surgical parameters, comorbidities (Table 1). The mean ages were similar between groups (Group O: 41.1 ± 11.8 years vs. Group S: 38.5 ± 11.5 years, *p* = 0.162). No statistically significant differences were observed between groups in ASA classification, smoking history, surgical history, or distribution of comorbidities such as hypertension and diabetes (all *p* > 0.05). The mean operative duration was also comparable between groups (Group O: 2.04 ± 0.79 h vs. Group S: 2.06 ± 0.71 h, *p* = 0.846).

**Table 1:** Baseline characteristics of the study population.

Characteristic	Group O (*n* = 76)	Group S (*n* = 76)	*p*-value
Age (years)	41.13 ± 11.80	38.47 ± 11.50	0.162
Height (cm)	157.79 ± 12.99	158.91 ± 5.50	0.491
Weight (kg)	61.01 ± 15.54	56.46 ± 8.84	0.028
BMI (kg/m^2^)	23.58 ± 3.90	22.36 ± 3.36	0.041
Surgery Duration (h)	2.04 ± 0.79	2.06 ± 0.71	0.846
ASA			0.699
I	30 (39.47%)	37 (48.68%)	
II	46 (60.53%)	39 (51.32%)	
Smoking history			0.316
No	76 (100.00%)	75 (98.68%)	
Yes	0 (0.00%)	1 (1.32%)	
Surgical history			0.509
No	29 (38.16%)	33 (43.42%)	
Yes	47 (61.84%)	43 (56.58%)	
Hypertension			0.512
No	62 (81.58%)	65 (85.53%)	
Yes	14 (18.42%)	11 (14.47%)	
DM			1.000
No	70 (92.11%)	70 (92.11%)	
Yes	6 (7.89%)	6 (7.89%)	

BMI, body mass index; ASA, American Society of Anesthesiologists; DM, Diabetes mellitus.

Notably, mean body weight was significantly higher in Group O than in Group S (61.0 ± 15.5 kg vs. 56.5 ± 8.8 kg, *p* = 0.028); body mass index (BMI), the core indicator reflecting nutritional status, also showed a statistically significant difference between groups (23.58 ± 3.90 kg/m^2^ vs. 22.36 ± 3.36 kg/m^2^, *p* = 0.041).

### Primary outcome: resting VAS pain scores

**Figure 2:**
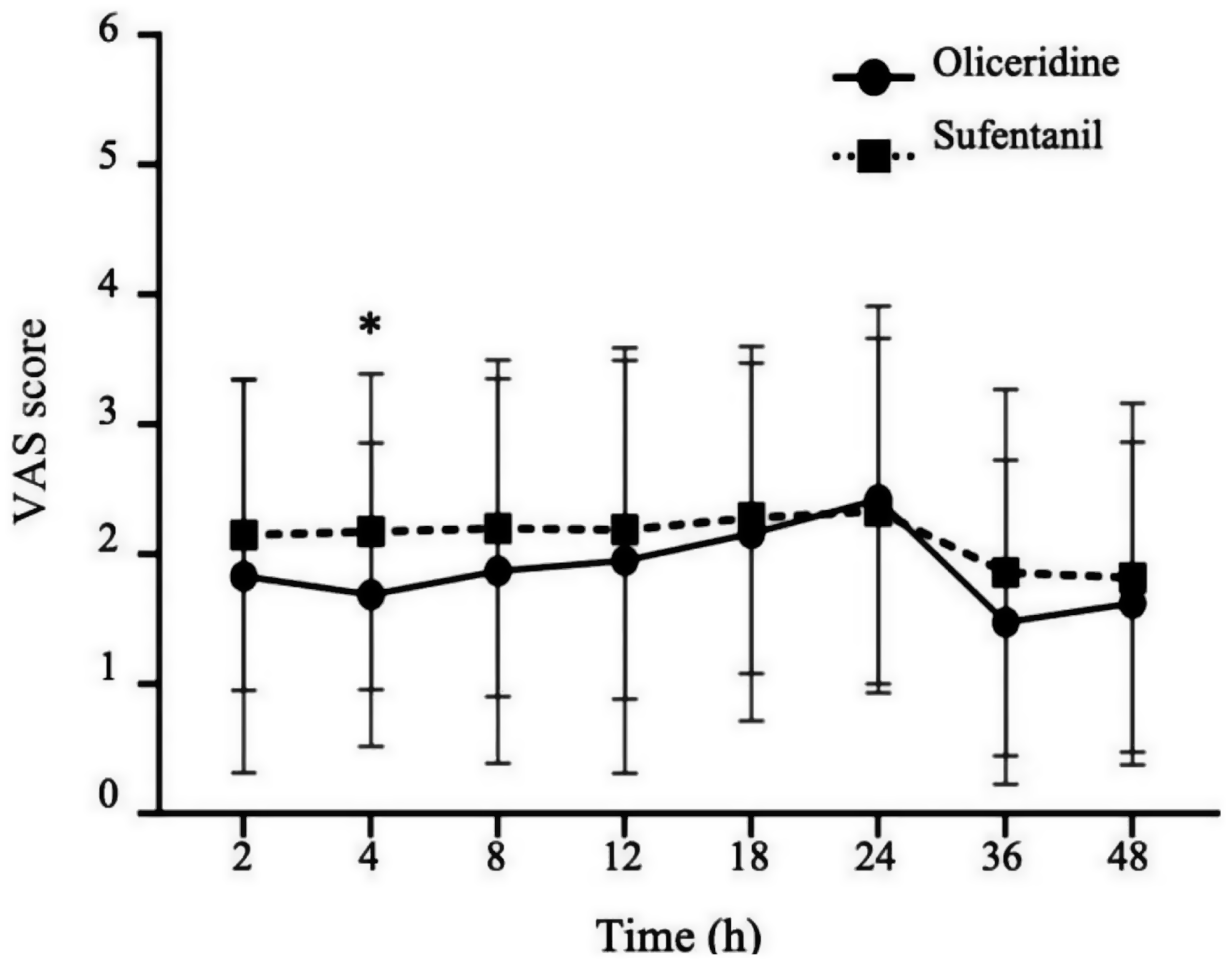
Comparison of postoperative resting VAS pain scores at 2, 4, 8, 12, 18, 24, 36, and 48 h after surgery between two groups. Data are presented as mean ± standard deviation. *p < 0.05 versus Group S. VAS, visual analog scale; Group O, Oliceridine group; Group S, Sufentanil group.

Figure 2 illustrates the trend of resting VAS scores over 48 h postoperatively in both groups. The VAS scores for both groups exhibited a decreasing trend from 2 to 48 h postoperatively. In the unadjusted analysis, Group O demonstrated significantly lower VAS scores at 4 h postoperatively compared to Group S (1.68 ± 1.17 vs. 2.17 ± 1.22, *p* = 0.013). However, after applying Bonferroni correction for the eight repeated measurements (adjusted significance threshold *α* = 0.05/8 = 0.00625), this difference no longer reached statistical significance. No significant differences in VAS scores between the two groups at other time points (2, 8, 12, 18, 24, 36, and 48 h postoperatively, all *p* > 0.05). Specifically, at 2 h postoperatively: Group O 1.83 ± 1.52 vs. Group S 2.14 ± 1.20 (*p* = 0.156); at 24 h postoperatively: Group O 2.42 ± 1.49 vs. Group S 2.33 ± 1.33 (*p* = 0.688); at 48 h postoperatively: Group O 1.62 ± 1.24 vs. Group S 1.82 ± 1.34 (*p* = 0.349). These results suggest generally comparable analgesic efficacy between oliceridine and sufentanil for resting pain control.

### Postoperative analgesic requirements and rescue analgesia

No significant difference was observed in the total number of PCIA demands within 48 h postoperatively between the two groups (Group O: 0.45 ± 1.12 vs. Group S: 0.86 ± 4.42, *p* = 0.437). Regarding rescue analgesia requirements, the proportion of patients requiring tramadol was similar between groups (Group O: 6.58% vs. Group S: 6.58%, *p* = 1.000), with no between-group difference in analgesic rescue. These results indicate that oliceridine and sufentanil exhibited no significant differences in patient-controlled analgesic demands or frequency of rescue analgesia use.

### Incidence of adverse effects

Regarding postoperative adverse events, the oliceridine and sufentanil groups demonstrated different incidence rates. PONV was the most common adverse event, occurring in 14 patients (18.4%) in the oliceridine group and 29 patients (38.2%) in the sufentanil group. The oliceridine group demonstrated a significantly lower risk of PONV compared with the sufentanil group (RR = 0.48, 95% CI (0.28–0.84), *p* = 0.007).

To determine whether the observed reduction in PONV with oliceridine was independent of baseline differences, we performed multivariable logistic regression adjusting for potential confounders. In the unadjusted model, oliceridine was associated with a significantly lower risk of PONV compared with sufentanil (OR 0.43, 95% CI 0.21–0.88, *p* = 0.021). After adjustment for age and BMI (Model I), the effect remained significant (OR 0.48, 95% CI 0.23–0.99, *p* = 0.047). Furthermore, after full adjustment for age, BMI, ASA status, smoking history, surgical history, hypertension, and diabetes (Model II), oliceridine continued to demonstrate a protective effect against PONV (OR 0.45, 95% CI 0.21–0.94, *p* = 0.034) (Supplementary Table S1).

For other adverse events, the incidence of dizziness was 31 cases (40.8%) in the oliceridine group and 37 cases (48.7%) in the sufentanil group, with no statistically significant between-group difference (RR = 0.84, 95% CI (0.59–1.20), *p* = 0.328). The incidence of fever was 7 cases (9.2%) in the oliceridine group and 3 cases (3.9%) in the sufentanil group (RR = 2.33, 95% CI (0.63–8.69), *p* = 0.191). The incidence of headache was 6 cases (7.9%) in the oliceridine group and 2 cases (2.6%) in the sufentanil group (RR = 3.00, 95% CI (0.63–14.40), *p* = 0.146).

Adverse events with lower incidence included: cough (Group O: 4 cases (5.3%) vs. Group S: 1 case (1.3%), RR = 4.00, 95% CI (0.46–34.97), *p* = 0.172), abdominal distension (Group O: 3 cases (4.0%) vs. Group S: 1 case (1.3%), RR = 3.00, 95% CI (0.32–28.20), *p* = 0.311), and pruritus (Group O: 0 cases (0.0%) vs. Group S: 1 case (1.3%), RR = 0.00, 95% CI (0.00–0.03), *p* = 0.316). None of these differences reached statistical significance (see Table 2).

**Table 2:** Comparison of postoperative adverse events between groups.

Adverse event	Group O (*n* = 76)	Group S (*n* = 76)	RR (95% CI)	*p*-value
PONV			0.48 (0.28–0.84)	0.007
No	62 (81.58%)	47 (61.84%)		
Yes	14 (18.42%)	29 (38.16%)		
Pruritus			0.00 (0.00–0.03)	0.316
No	76 (100.00%)	75 (98.68%)		
Yes	0 (0.00%)	1 (1.32%)		
Fever			2.33 (0.63–8.69)	0.191
No	69 (90.79%)	73 (96.05%)		
Yes	7 (9.21%)	3 (3.95%)		
Headache			3.00 (0.63–14.40)	0.146
No	70 (92.11%)	74 (97.37%)		
Yes	6 (7.89%)	2 (2.63%)		
Dizziness			0.84 (0.59–1.20)	0.328
No	45 (59.21%)	39 (51.32%)		
Yes	31 (40.79%)	37 (48.68%)		
Cough			4.00 (0.46–34.97)	0.172
No	72 (94.74%)	75 (98.68%)		
Yes	4 (5.26%)	1 (1.32%)		
Abdominal distension			3.00 (0.32–28.20)	0.311
No	73 (96.05%)	75 (98.68%)		
Yes	3 (3.95%)	1 (1.32%)		

PONV, Postoperative nausea and vomiting; Group O, oliceridine group; Group S, sufentanil group.

### Postoperative gastrointestinal function recovery

The two groups showed no significant overall differences in postoperative gastrointestinal function recovery indicators (Table 3); however, regarding time to first flatus, Group S demonstrated a shorter time compared with Group O.

**Table 3:** Comparison of postoperative gastrointestinal recovery between groups.

Gastrointestinal recovery parameter	Group O (*n* = 76)	Group S (*n* = 76)	*p*-value
First flatus occurrence			0.147^a^
No	4 (5.26%)	9 (11.84%)	
Yes	72 (94.74%)	67 (88.16%)	
First defecation occurrence			0.656^a^
No	65 (85.53%)	63 (82.89%)	
Yes	11 (14.47%)	13 (17.11%)	
Time to first flatus (h)	27.16 ± 9.33	23.23 ± 9.36	0.014^b^
Time to first defecation (h)	42.18 ± 5.13	39.21 ± 6.72	0.243^b^

Data are presented as number (percentage) or mean ± standard deviation.

a Chi-square test or Fisher’s exact test (for categorical variables).

b Independent samples *t*-test (for continuous variables).

For time to first flatus, 4 patients (5.3%) in Group O and 9 patients (11.8%) in Group S had not experienced flatus within 48 h postoperatively; the remaining patients recovered flatus within 48 h, with 72 cases (94.7%) in Group O and 67 cases (88.2%) in Group S. The mean time to first flatus was 27.16 ± 9.33 h in Group O and 23.23 ± 9.36 h in Group S, with a mean difference of 3.93 h (95% CI: 0.79–7.07, *p* = 0.014 by independent samples *t*-test), indicating significantly shorter time to first flatus in Group S.

**Figure 3:**
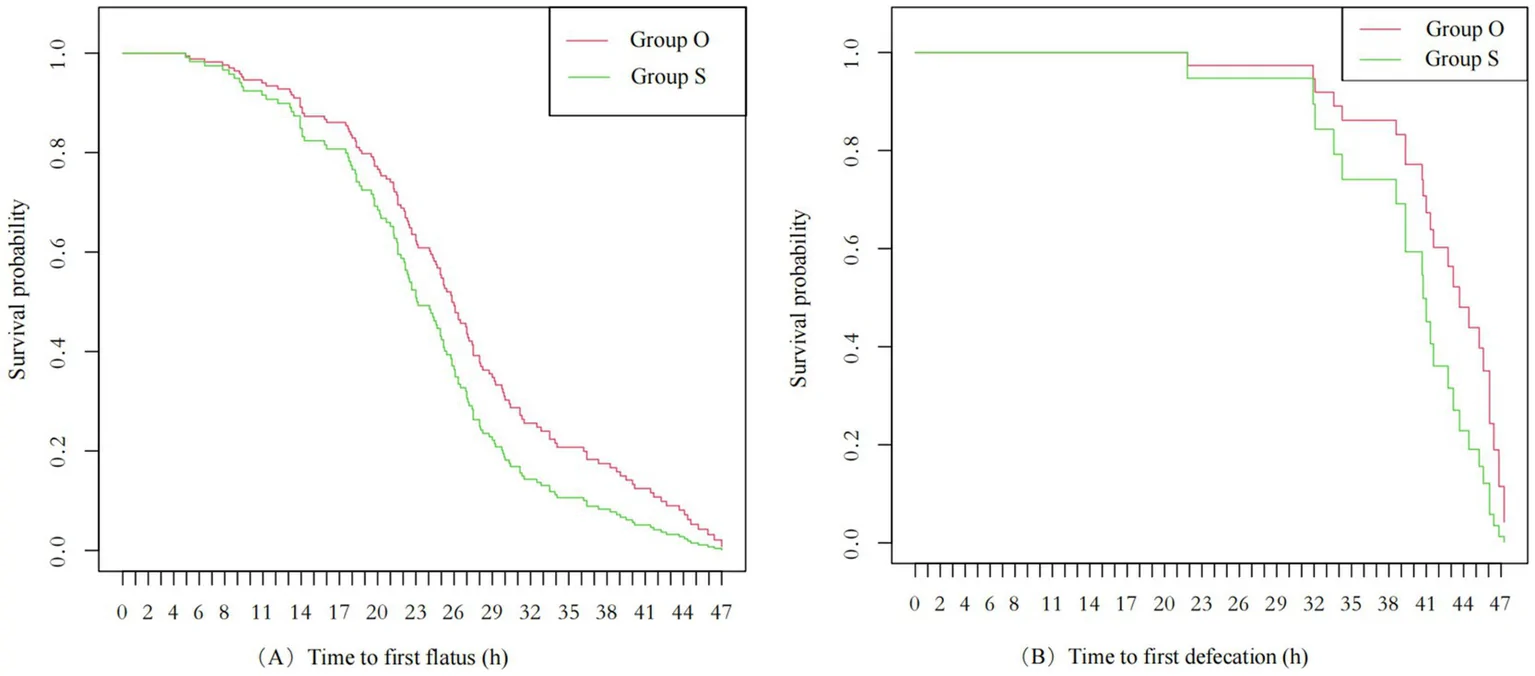
Kaplan–Meier analysis of time to first flatus and defecation. **(A)** Time to first flatus. The red line represents the oliceridine group (Group O), and the green line represents the sufentanil group (Group S). There was a statistically significant difference between groups (*p* = 0.023, log-rank test). **(B)** Time to first defecation. The red line represents the oliceridine group (Group O), and the green line represents the sufentanil group (Group S). There was no statistically significant difference between groups (*p* = 0.656, log-rank test). Group O, oliceridine group; Group S, sufentanil group.

Figure 3 presents the Kaplan–Meier analysis of time to first flatus and defecation. Figure 3A demonstrates that Group S exhibited a higher cumulative incidence of flatus in the early postoperative period (approximately 20–30 h), with overall curve distribution trends consistent with log-rank test results (*p* = 0.023), further supporting faster flatus recovery in Group S.

For time to first defecation, 65 patients (85.5%) in Group O and 63 patients (82.9%) in Group S had not defecated within 48 h postoperatively; patients who defecated within 48 h numbered 11 (14.5%) in Group O and 13 (17.1%) in Group S. The mean time to first defecation was 42.18 ± 5.13 h in Group O and 39.21 ± 6.72 h in Group S, with a mean difference of 2.97 h (95% CI: −1.97–7.91, *p* = 0.243), indicating no statistically significant difference between groups.

Figure 3B Presents the Kaplan–Meier survival curves for time to first defecation. The two curves are relatively close, particularly after 30 h postoperatively, with almost overlapping cumulative defecation incidence (log-rank *p* = 0.656), suggesting no significant difference in defecation recovery between groups and further supporting the statistical analysis results.

### Patient satisfaction

Regarding postoperative satisfaction, patient satisfaction scores in Group O were 4.09 ± 0.81, compared with 3.74 ± 0.72 in Group S. The Cohen’s d between groups was 0.45 (95% CI: 0.13–0.77, *p* = 0.006). These results indicate that postoperative satisfaction was significantly higher in Group O than in Group S.

### Postoperative cognitive function

No significant differences were observed in MMSE scores between the two groups at 24 or 48 h postoperatively. At 24 h postoperatively: Group O 25.7 ± 2.3 vs. Group S 25.8 ± 2.5 (*p* = 0.792); at 48 h postoperatively: Group O 27.0 ± 2.1 vs. Group S 27.0 ± 2.1 (*p* = 0.877). No cases of POCD occurred in either group.

## Discussion

This single-center, randomized, double-blind trial provides the first direct comparison of oliceridine versus sufentanil for postoperative PCIA in Chinese patients undergoing gynecological laparoscopic surgery. The principal findings are: (1) oliceridine provided comparable analgesic efficacy to sufentanil at most postoperative time points, with comparable PCIA demands and rescue analgesia requirements; (2) PONV incidence was significantly lower with oliceridine (19.48% vs. 36.14%, *p* = 0.019) despite higher baseline BMI in this group, and this protective effect remained significant after adjustment for potential confounders; (3) patient satisfaction was significantly higher with oliceridine (*p* = 0.006); and (4) time to first flatus was prolonged in the oliceridine group, though cognitive function and other adverse events were similar between groups.

Our findings align with prior evidence on oliceridine. Phase III trials demonstrated non-inferior analgesia to morphine (26, 27), with the APOLLO-2 trial and subsequent pooled analyses further confirming reduced PONV and respiratory depression risks (27, 28). Pooled analyses further confirmed improved gastrointestinal tolerability compared with morphine (28). Direct comparisons with sufentanil are limited but supportive: Duan et al. (29) reported superior recovery quality and fewer adverse events with oliceridine following total laparoscopic hysterectomy, while Na et al. (30) observed faster early recovery after thoracoscopic surgery. These studies corroborate our conclusion that oliceridine maintains equivalent analgesia while improving safety and patient-reported outcomes.

The favorable profile of oliceridine likely reflects its biased *μ*-opioid receptor agonism. Selective G-protein pathway activation produces analgesia while minimizing *β*-arrestin recruitment, which mediates opioid-related nausea, vomiting, and respiratory depression (16, 17). Clinical and pharmacokinetic studies have consistently shown reduced gastrointestinal adverse effects and less severe respiratory depression with oliceridine compared with traditional opioids (19, 20, 28, 31, 32). Although debate exists regarding whether this profile stems from true biased agonism or low intrinsic efficacy (33, 34), the clinical benefits remain evident regardless of mechanism.

These results carry important practical implications. Female patients represent a high-risk PONV population, with rates often exceeding 35% with conventional opioids (13, 15). The nearly 50% reduction in PONV with oliceridine—observed despite higher baseline BMI—supports its value in this demographic and aligns with ERAS goals of minimizing complications and accelerating recovery (8, 35–37). Although time to first flatus was delayed in the oliceridine group, patient satisfaction remained significantly higher, suggesting that the subjective experience of reduced nausea outweighs this isolated objective difference. This is consistent with reports of improved postoperative recovery quality and sleep with oliceridine (25, 38).

This study has limitations. First, its single-center design may limit generalizability. Second, the sample size, while adequate for the primary efficacy endpoint, may be insufficient for rare adverse events such as severe respiratory depression. Third, the 48-h follow-up captures the peak PONV period but precludes assessment of chronic postoperative pain or long-term functional outcomes. Fourth, baseline weight and BMI differed significantly between groups. While this imbalance is a limitation, the relationship between BMI and PONV remains controversial: the Apfel score does not include BMI (13), and consensus guidelines classify it as a factor with limited or disproven association (15). Some studies reported no independent association (39), whereas others suggested higher BMI might be protective (40, 41)or, conversely, a potential risk factor according to other reports (42). Consequently, the direction of any confounding is uncertain—if higher BMI were a risk factor, the observed PONV reduction with oliceridine would represent a conservative benefit; if protective, the effect might be exaggerated. Importantly, multivariable adjustment for BMI confirmed the robustness of our primary finding (adjusted OR = 0.45, 95% CI 0.21–0.94, *p* = 0.034). Finally, systematic respiratory monitoring was not performed, though no intervention-requiring respiratory depression was observed.

Future research should include multicenter trials to validate these findings, studies in special populations (elderly, obese, or respiratory-compromised patients), longer follow-up to assess chronic pain outcomes, and pharmacoeconomic analyses to evaluate cost-effectiveness. Further investigation into the differential effects of biased agonists on upper versus lower gastrointestinal motility is also warranted.

## Conclusion

In summary, for postoperative analgesia following gynecological laparoscopic surgery, oliceridine injection provides effective analgesia comparable to sufentanil injection while significantly reducing the risk of typical opioid-related adverse effects, particularly PONV, demonstrating an improved safety profile. Oliceridine represents an excellent alternative option for postoperative PCIA, especially suitable for high-risk PONV populations such as female patients. The results of this study provide important evidence-based medical evidence for the selection of clinical postoperative analgesic protocols.

## Data Availability

The raw data supporting the conclusions of this article are not publicly available due to ethical restrictions and patient privacy protection. Access to the data can be requested from the corresponding authors (Ronghua Huang, rhhuangsumc@163.com; Weiqi Ke, wqke2@stu.edu.cn) upon reasonable request and with appropriate ethical approval.
