# Supplementary material for: Efficacy and safety of oliceridine versus sufentanil for patient-controlled intravenous analgesia following gynecological laparoscopic surgery: a randomized, double-blind, parallel-group trial
Source: Front Med (Lausanne). 2026 Aug 11;13:1866393. doi: 10.3389/fmed.2026.1866393 (PMC13503371; doi:10.3389/fmed.2026.1866393)
Supplement: Supplementary file 1 [file Table_1.DOCX]

**Table S1. Multivariable Logistic Regression Analysis for Postoperative Nausea and Vomiting (PONV)**

|  | Crude Model | | Model I | | Model II | |
| --- | --- | --- | --- | --- | --- | --- |
|  | OR (95%CI) | P -value | OR (95%CI) | P -value | OR (95%CI) | P -value |
| Sufentanil | Reference | | Reference | | Reference | |
| Oliceridine | 0.43 (0.21, 0.88) | 0.0207 | 0.48 (0.23, 0.99) | 0.0465 | 0.45 (0.21, 0.94) | 0.0344 |

The Crude model was unadjusted for any factors.

Model I adjusted for age and BMI.

Model II adjusted for age, BMI, ASA classification, smoking history, surgical history, hypertension, and diabetes.
